# Supplementary material for: Unveiling the Bioactive Potential of the Invasive Jellyfish Phyllorhiza punctata Through Integrative Transcriptomic and Proteomic Analyses
Source: Biomolecules. 2025 Aug 4;15(8):1121. doi: 10.3390/biom15081121 (PMC12383608; doi:10.3390/biom15081121)
Supplement: Supplementary file 1 [file biomolecules-15-01121-s001.zip › Suplementary Material/FileS3/PEPFOLD4/XP_065059929_5019/clusters.html]

 PEP-FOLD clusters


Download archive of all models  here   

|  |  |  |
| --- | --- | --- |
| # | file | sOPEP |
| # cluster 1 - Size: 1 |  |  |
|  | XP\_065059929\_5019-model1 | -23.5852 |
| # cluster 2 - Size: 2 |  |  |
|  | XP\_065059929\_5019-model2 | -23.3048 |
|  | XP\_065059929\_5019-model2.1 | -22.392 |
| # cluster 3 - Size: 2 |  |  |
|  | XP\_065059929\_5019-model3 | -22.8082 |
|  | XP\_065059929\_5019-model3.1 | -20.0964 |
| # cluster 4 - Size: 2 |  |  |
|  | XP\_065059929\_5019-model4 | -22.6123 |
|  | XP\_065059929\_5019-model4.1 | -16.6178 |
| # cluster 5 - Size: 3 |  |  |
|  | XP\_065059929\_5019-model5 | -21.4573 |
|  | XP\_065059929\_5019-model5.1 | -21.4185 |
|  | XP\_065059929\_5019-model5.2 | -18.9806 |
| # cluster 6 - Size: 1 |  |  |
|  | XP\_065059929\_5019-model6 | -19.6018 |
| # cluster 7 - Size: 1 |  |  |
|  | XP\_065059929\_5019-model7 | -19.3779 |
| # cluster 8 - Size: 1 |  |  |
|  | XP\_065059929\_5019-model8 | -18.8958 |
| # cluster 9 - Size: 2 |  |  |
|  | XP\_065059929\_5019-model9 | -17.59 |
|  | XP\_065059929\_5019-model9.1 | -16.9387 |
| # cluster 10 - Size: 2 |  |  |
|  | XP\_065059929\_5019-model10 | -16.9403 |
|  | XP\_065059929\_5019-model10.1 | -16.122 |
| # cluster 11 - Size: 2 |  |  |
|  | XP\_065059929\_5019-model11 | -16.7234 |
|  | XP\_065059929\_5019-model11.1 | -15.6887 |
| # cluster 12 - Size: 1 |  |  |
|  | XP\_065059929\_5019-model12 | -16.4136 |
| # cluster 13 - Size: 1 |  |  |
|  | XP\_065059929\_5019-model13 | -16.2557 |
| # cluster 14 - Size: 3 |  |  |
|  | XP\_065059929\_5019-model14 | -16.1243 |
|  | XP\_065059929\_5019-model14.1 | -15.3531 |
|  | XP\_065059929\_5019-model14.2 | -13.2384 |
| # cluster 15 - Size: 1 |  |  |
|  | XP\_065059929\_5019-model15 | -16.0566 |
| # cluster 16 - Size: 1 |  |  |
|  | XP\_065059929\_5019-model16 | -16.0495 |
| # cluster 17 - Size: 2 |  |  |
|  | XP\_065059929\_5019-model17 | -15.4033 |
|  | XP\_065059929\_5019-model17.1 | -14.8361 |
| # cluster 18 - Size: 4 |  |  |
|  | XP\_065059929\_5019-model18 | -15.3235 |
|  | XP\_065059929\_5019-model18.1 | -13.4153 |
|  | XP\_065059929\_5019-model18.2 | -13.2516 |
|  | XP\_065059929\_5019-model18.3 | -11.91 |
| # cluster 19 - Size: 1 |  |  |
|  | XP\_065059929\_5019-model19 | -15.2346 |
| # cluster 20 - Size: 1 |  |  |
|  | XP\_065059929\_5019-model20 | -15.1638 |
| # cluster 21 - Size: 1 |  |  |
|  | XP\_065059929\_5019-model21 | -14.6759 |
| # cluster 22 - Size: 4 |  |  |
|  | XP\_065059929\_5019-model22 | -14.5933 |
|  | XP\_065059929\_5019-model22.1 | -14.2175 |
|  | XP\_065059929\_5019-model22.2 | -13.6533 |
|  | XP\_065059929\_5019-model22.3 | -11.1807 |
| # cluster 23 - Size: 1 |  |  |
|  | XP\_065059929\_5019-model23 | -14.3868 |
| # cluster 24 - Size: 1 |  |  |
|  | XP\_065059929\_5019-model24 | -14.2694 |
| # cluster 25 - Size: 2 |  |  |
|  | XP\_065059929\_5019-model25 | -14.1034 |
|  | XP\_065059929\_5019-model25.1 | -11.739 |
| # cluster 26 - Size: 2 |  |  |
|  | XP\_065059929\_5019-model26 | -13.9021 |
|  | XP\_065059929\_5019-model26.1 | -12.1387 |
| # cluster 27 - Size: 2 |  |  |
|  | XP\_065059929\_5019-model27 | -13.8795 |
|  | XP\_065059929\_5019-model27.1 | -12.6994 |
| # cluster 28 - Size: 1 |  |  |
|  | XP\_065059929\_5019-model28 | -13.7757 |
| # cluster 29 - Size: 2 |  |  |
|  | XP\_065059929\_5019-model29 | -13.6935 |
|  | XP\_065059929\_5019-model29.1 | -10.685 |
| # cluster 30 - Size: 2 |  |  |
|  | XP\_065059929\_5019-model30 | -13.6031 |
|  | XP\_065059929\_5019-model30.1 | -12.3422 |
| # cluster 31 - Size: 1 |  |  |
|  | XP\_065059929\_5019-model31 | -13.5279 |
| # cluster 32 - Size: 2 |  |  |
|  | XP\_065059929\_5019-model32 | -13.5031 |
|  | XP\_065059929\_5019-model32.1 | -12.2863 |
| # cluster 33 - Size: 1 |  |  |
|  | XP\_065059929\_5019-model33 | -13.3482 |
| # cluster 34 - Size: 5 |  |  |
|  | XP\_065059929\_5019-model34 | -13.2081 |
|  | XP\_065059929\_5019-model34.1 | -13.1412 |
|  | XP\_065059929\_5019-model34.2 | -12.6818 |
|  | XP\_065059929\_5019-model34.3 | -12.6183 |
|  | XP\_065059929\_5019-model34.4 | -12.144 |
| # cluster 35 - Size: 2 |  |  |
|  | XP\_065059929\_5019-model35 | -13.1096 |
|  | XP\_065059929\_5019-model35.1 | -12.7992 |
| # cluster 36 - Size: 1 |  |  |
|  | XP\_065059929\_5019-model36 | -12.8958 |
| # cluster 37 - Size: 2 |  |  |
|  | XP\_065059929\_5019-model37 | -12.8343 |
|  | XP\_065059929\_5019-model37.1 | -11.6298 |
| # cluster 38 - Size: 1 |  |  |
|  | XP\_065059929\_5019-model38 | -12.78 |
| # cluster 39 - Size: 2 |  |  |
|  | XP\_065059929\_5019-model39 | -12.7501 |
|  | XP\_065059929\_5019-model39.1 | -11.8358 |
| # cluster 40 - Size: 1 |  |  |
|  | XP\_065059929\_5019-model40 | -12.7215 |
| # cluster 41 - Size: 4 |  |  |
|  | XP\_065059929\_5019-model41 | -12.709 |
|  | XP\_065059929\_5019-model41.1 | -12.5981 |
|  | XP\_065059929\_5019-model41.2 | -12.1902 |
|  | XP\_065059929\_5019-model41.3 | -11.9751 |
| # cluster 42 - Size: 2 |  |  |
|  | XP\_065059929\_5019-model42 | -12.6902 |
|  | XP\_065059929\_5019-model42.1 | -11.7853 |
| # cluster 43 - Size: 1 |  |  |
|  | XP\_065059929\_5019-model43 | -12.5424 |
| # cluster 44 - Size: 1 |  |  |
|  | XP\_065059929\_5019-model44 | -12.48 |
| # cluster 45 - Size: 3 |  |  |
|  | XP\_065059929\_5019-model45 | -12.4282 |
|  | XP\_065059929\_5019-model45.1 | -11.8595 |
|  | XP\_065059929\_5019-model45.2 | -11.398 |
| # cluster 46 - Size: 1 |  |  |
|  | XP\_065059929\_5019-model46 | -12.4024 |
| # cluster 47 - Size: 1 |  |  |
|  | XP\_065059929\_5019-model47 | -12.4021 |
| # cluster 48 - Size: 1 |  |  |
|  | XP\_065059929\_5019-model48 | -12.1601 |
| # cluster 49 - Size: 1 |  |  |
|  | XP\_065059929\_5019-model49 | -12.0443 |
| # cluster 50 - Size: 1 |  |  |
|  | XP\_065059929\_5019-model50 | -11.9463 |
| # cluster 51 - Size: 2 |  |  |
|  | XP\_065059929\_5019-model51 | -11.8904 |
|  | XP\_065059929\_5019-model51.1 | -10.8561 |
| # cluster 52 - Size: 1 |  |  |
|  | XP\_065059929\_5019-model52 | -11.8545 |
| # cluster 53 - Size: 1 |  |  |
|  | XP\_065059929\_5019-model53 | -11.6748 |
| # cluster 54 - Size: 2 |  |  |
|  | XP\_065059929\_5019-model54 | -11.5403 |
|  | XP\_065059929\_5019-model54.1 | -10.9938 |
| # cluster 55 - Size: 1 |  |  |
|  | XP\_065059929\_5019-model55 | -11.4629 |
| # cluster 56 - Size: 1 |  |  |
|  | XP\_065059929\_5019-model56 | -11.3562 |
| # cluster 57 - Size: 1 |  |  |
|  | XP\_065059929\_5019-model57 | -11.2646 |
| # cluster 58 - Size: 1 |  |  |
|  | XP\_065059929\_5019-model58 | -11.1063 |
| # cluster 59 - Size: 1 |  |  |
|  | XP\_065059929\_5019-model59 | -11.1019 |
| # cluster 60 - Size: 1 |  |  |
|  | XP\_065059929\_5019-model60 | -10.9792 |
| # cluster 61 - Size: 1 |  |  |
|  | XP\_065059929\_5019-model61 | -10.877 |
| # cluster 62 - Size: 1 |  |  |
|  | XP\_065059929\_5019-model62 | -10.3169 |
|  |  |  |
